# Pocapavir for neonatal enteroviral sepsis: limitations of rapid viral sequencing on treatment decisions

**DOI:** 10.1128/asmcr.00008-25

**Published:** 2025-07-08

**Authors:** Nathan L'Etoile, Sesh A. Sundararaman, Erin Theiller, Justin J. Miller, Ahmed M. Moustafa, Andrew P. Steenhoff, Audrey R. Odom John

**Affiliations:** 1 Children’s Hospital of Philadelphia, Philadelphia, Pennsylvania, USA; 2 Department of Pediatrics, Perelman School of Medicine, University of Pennsylvania 6572, Philadelphia, Pennsylvania, USA; 3 Department of Biochemistry and Biophysics, University of Pennsylvania 6572, Philadelphia, Pennsylvania, USA; Pattern Bioscience, Austin, Texas, USA

**Keywords:** enterovirus, investigational drug, whole genome sequencing, pocapavir, antiviral resistance

## Abstract

**Background:**

Neonatal enterovirus infection can be severe and life-threatening, and there is ongoing interest in the development of antiviral compounds; however, their clinical efficacy has not been definitively proven.

**Case Summary:**

We describe a case of neonatal enterovirus infection treated with the investigational therapeutic pocapavir, a viral capsid inhibitor. We describe the medical decision-making regarding the choice to offer pocapavir therapy. Because of clinical uncertainty about whether pocapavir contributed to clinical improvement, we performed sequencing of the viral genome and modeled the pocapavir pocket-binding site for evidence of genetic and protein changes before or after the initiation of pocapavir that might confer antiviral resistance.

**Conclusion:**

This case and our findings reveal the challenges in interpreting sequence analysis of clinical enterovirus isolates to inform clinical decision-making regarding the use of pocapavir for the treatment of neonatal enterovirus.

## INTRODUCTION

Neonatal enterovirus infection can be severe, with multi-organ dysfunction including myocarditis and hepatitis (1). Treatment is a challenge, as the few therapeutic options are supported by limited data (2). The antiviral capsid inhibitor pocapavir has shown some promise against neonatal enteroviral infection (3–5). While initially developed for poliovirus (6), pocapavir has been used investigationally in the treatment of neonatal enterovirus sepsis (3–5). Because pocapavir prevents viral uncoating, efficacy may decrease late in infection, and questions remain about potential resistance (6). We describe a case of severe neonatal enterovirus treated with pocapavir, presenting the clinical and virologic response following pocapavir administration and the results of virus sequencing.

## CASE PRESENTATION

The patient was an infant male born at 36 weeks via cesarean section for placenta previa to a family without significant medical/genetic history. During the third trimester of pregnancy, his mother had a cold-like illness (without viral testing). The immediate postnatal course included a sepsis evaluation with ampicillin and gentamicin (dosing unknown) for 48 hours, as well as nasogastric feeding due to feeding difficulty. At 6 days of life and while still admitted, the patient developed hypothermia, lethargy, and blood-streaked stools (day of illness [DOI] 1). He had direct hyperbilirubinemia, coagulopathy, and hepatocellular injury, with an aspartate aminotransferase (AST) of 10,690 units/L (reference range: ≤41 U/L) and alanine aminotransferase (ALT) of 1,203 units/L (reference range: ≤42 U/L). Nasopharyngeal viral PCR was positive for rhinovirus/enterovirus (Biofire Respiratory Panel). Given worsening coagulopathy, he was transferred to our institution on DOI 2.

**Fig 1:**
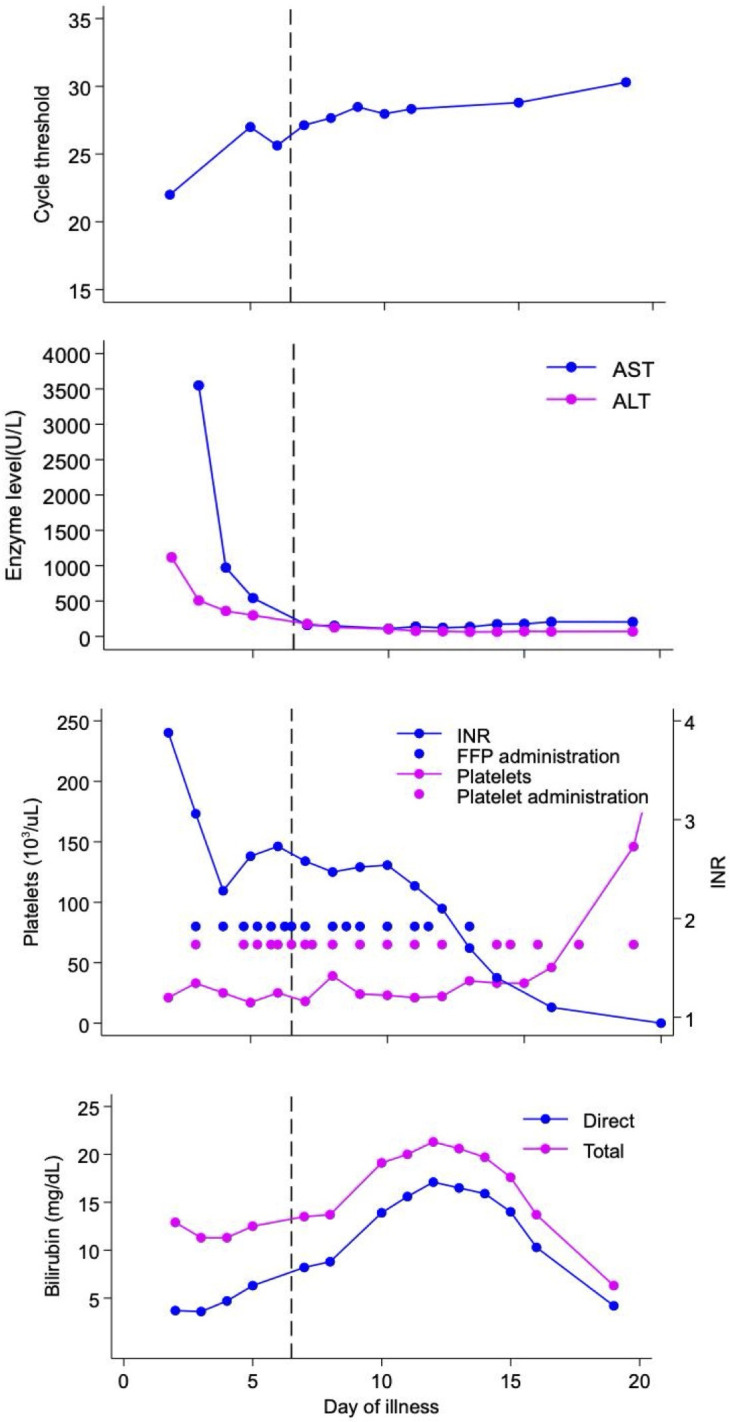
Graphical representation of the patient’s clinical course. Pocapavir was initiated on DOI 6, noting CT by DOI without substantial change after pocapavir; platelet count (reference range: 150–450 × 10^3^ /µL) and international normalized ratio (INR; reference range: ≤3.00) with unconnected dots denoting transfusions; AST and ALT during course of illness; and total and direct bilirubin (reference ranges: total bilirubin 0.6–1.4 mg/dL; direct bilirubin ≤0.3 mg/dL) during course of illness.

Upon arrival, the infant was somnolent but neurologically intact, with increased work of breathing requiring supplemental oxygen. He received 48 hours of vancomycin (15 mg/kg every 8 hours), cefotaxime (50 mg/kg every 8 hours), and acyclovir (20 mg/kg every 8 hours), and serum enterovirus PCR was positive with a cycle threshold (CT) of 22 (Fig. 1). Transfusions of fresh frozen plasma and platelets were required, and his troponin was elevated without echocardiographic evidence of cardiac failure. Due to anasarca, intravenous immune globulin (IVIG) was deferred. On DOI 6, in consultation with the family, enteral pocapavir (120 mg every 24 hours) was initiated for a total of 14 days. By treatment day 3 (DOI 8), the patient required fewer transfusions, and hepatitis began to resolve (Fig. 1). By DOI 11, parenteral nutrition was discontinued, and on DOI 15, he was weaned to room air. Virologic response to therapy was monitored by following the CT of serum enterovirus PCR as a proxy for viral load. The CT was defined as the number of replication cycles required to detect enterovirus RNA by PCR. Enterovirus PCR at our institution is a lab-developed multi-plex real-time PCR using published primers adapted for use in a TaqMan assay (7). As the patient was clinically improving despite a lack of substantial change in the CT values, IVIG was again deferred. By DOI 20, enterovirus PCR remained positive, and CT values remained largely unchanged, prompting interest in genomic evaluation for resistance.

Clinical monitoring included a 24-hour electroencephalogram on DOI 8 without seizure activity, an unremarkable head ultrasound, troponin undetectable by DOI 11, and normal echocardiograms. The patient never developed bleeding. Prior to discharge, he required a continuous glucose infusion for hypoglycemia and ursodiol for conjugated hyperbilirubinemia (Fig. 1). The total admission duration was 1 month from birth. At 5 months of age, his liver dysfunction had resolved, a hearing screen was normal, and there was no documented developmental delay.

*In vitro* and *in vivo* studies have demonstrated that enteroviruses can develop resistance to pocapavir and other viral capsid-targeting compounds within 2 days of initiating therapy (6). Most resistance mutations occur near the drug-binding pocket of the VP1 capsid protein (I99F in rhinovirus 2; A150T/V, C199R/Y, V188I, and E276 in rhinovirus 14; M252L and A156T in enterovirus D-68; or I194F/M in poliovirus 1), although a single mutation (A24V) in the VP3 capsid protein has been observed in poliovirus 1 (6, 8, 9). We queried whether the lack of clear clinical or virologic response to pocapavir might be due to preexisting or newly developed genetic variation that conferred pocapavir resistance. We extracted and sequenced enterovirus RNA from serum samples collected on DOI 6 (before initiation of pocapavir) and DOI 13 (7 days after initiation). Total RNA was extracted (Qiagen RNeasy), enriched for enterovirus RNA (Illumina Viral Surveillance Panel v2 kit), and sequenced (Illumina MiSeq; 2 × 150 bp paired-end reads). Quality control, adapter trimming, and human read filtering were performed (Sunbeam) (10), and genomic sequences for the patient’s isolate were generated via *de novo* assembly with iterative read mapping in Geneious Prime v2024.0.4 (Biomatters, Auckland, New Zealand). *De novo* assembly parameters included word length 14, index word length 12, max gap size 2, max gap per read 15%, max mismatches per read 30%, and max ambiguity 4. Contigs that matched enterovirus sequences were identified by nucleotide blast, aligned to the closest enterovirus reference using the MUSCLE v5.1 PPP algorithm, and manually curated to produce a single working reference sequence (11). Reads from pre- and post-pocapavir samples were then mapped iteratively (five iterations) to this working reference using the same mapping parameters except for a max gap size of 50. Final pre- and post-pocapavir consensus sequences were then generated using samtools consensus v1.19.2 (Bayesian consensus calling algorithm, min read depth: 5 and min consensus base quality: 40) (12). This approach generated a genome for our patient’s isolate with full coverage of the protein-coding region (pre-treatment sample: 96× average depth, 64× average depth across VP1; post-treatment sample: 59× average depth, and 37× average depth across VP1).

**Fig 2:**
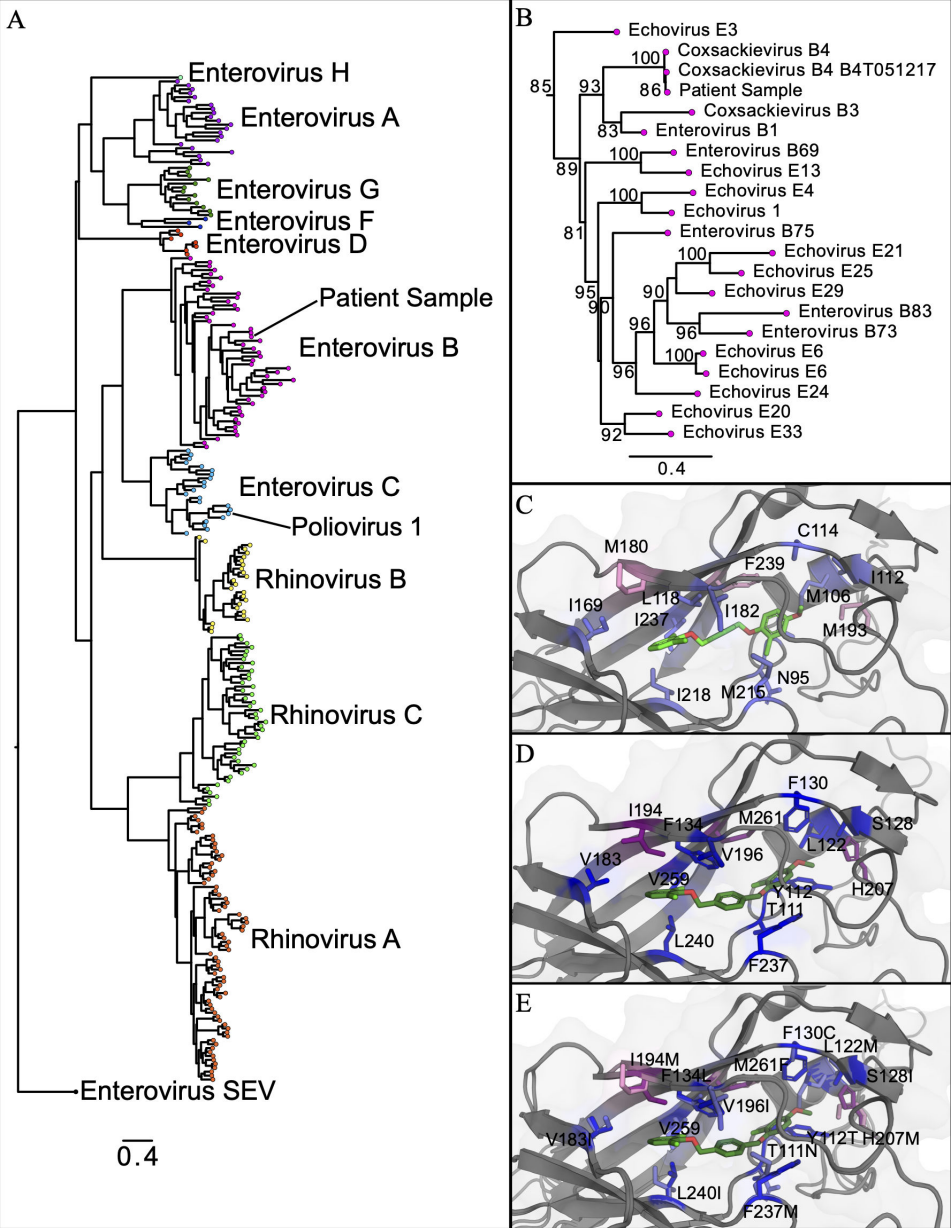
Phylogenetic and *in silico* characterization of patient enterovirus isolate. (**A**) Phylogenetic analysis of VP1 capsid protein sequences from the patient’s enterovirus and reference sequences. (**B**) Expanded phylogeny of closely related sequences. Numbers at internal nodes represent bootstrap support values (only numbers >80% are shown). (**C–E**) Molecular docking of pocapavir (green) to the predicted VP1 structure of the patient’s isolate (**C**) and poliovirus 1 VP1 (1eah) (**D**). (**E**) Sites of amino acid variation in VP1 between reference poliovirus 1 (darker) and patient enterovirus isolate (lighter). Blues indicate residues that are distinct between poliovirus and patient VP1, and reds are positions associated with resistance to pocapavir in poliovirus. Phylogenies were generated using RAxML-NG v1.2.2 with 100 bootstraps. Substitution models were determined by ModelTest-NG v0.1.7 (13, 14).

Phylogenetic analysis identified the patient’s isolate as coxsackievirus B4 (Fig. 2A). Comparisons of pre- and post-treatment viral sequences did not identify any nucleotide changes in VP1 or VP3, strongly suggesting that resistance did not emerge during treatment.

A prior study showed that the majority of coxsackie B4 strains were susceptible to pocapavir *in vitro* (IC50s: 30–900 ng/mL) (15); however, these strains were not sequenced. We sought to use *in silico* analyses to evaluate whether the patient’s virus possessed genetic evidence of reduced pocapavir sensitivity. We focused on VP1 and the pocapavir binding pocket, first generating a three-dimensional model of the patient isolate VP1 using the structure of pocapavir-bound poliovirus 2 VP1 (PDB 1eah) as a template (Modeller v10.2) (16, 17). Using AutoDock Vina, we docked pocapavir into the binding pockets of the patient isolate, coxsackie B4 virus, and murine poliovirus VP1s (18). Docking was performed using a 14 × 14 × 20 box centered on the 1eah pocapavir-binding pocket, requesting 20 binding modes, with an exhaustiveness of 128, with 10 replicas per run. While pocapavir fits each pocket, the murine poliovirus VP1 has the highest affinity, and coxsackie B4 has the lowest affinity (−11.2 ± 0, −10.0 ± 0, and –8.69 ± 0.03 kcal/mol). Notably, pocapavir docks in a slightly different conformation for patient VP1 compared to the reference crystal structure (Fig. 2B and C) (13, 14).

While poliovirus 1 and the patient isolate VP1s are similar (49% identical and 55% similar), their binding pockets differ greatly (Fig. 2D). The patient isolate VP1 has several amino acid changes that significantly impact binding pocket chemistry (e.g., Y112T, L122M, S128I, F134L, and V183I) and include several variants associated with poliovirus pocapavir resistance (e.g., I194M, H207M, and M261F; Fig. 2D). However, alignment (MUSCLE v5.1 PPP algorithm) (11) and comparison (AliView v1.28) (19) with publicly available coxsackievirus B4 VP1 sequences (*n* = 227) showed that these amino acids are highly conserved across the lineage (Table 1). We, therefore, predict that our patient’s isolate is likely pocapavir susceptible, as per previously characterized B4 strains.

**TABLE 1:** Sequence conservation of pocapavir binding pocket in the patient’s isolate

Residue in poliovirus 1	Residue in patient sequence	Percent of publicly available coxsackievirus B4 sequences with identical amino acid
VAL87	VAL73	100.0
TRP108	TRP92	100.0
ILE10	ILE94	100.0
THR111	ASN95	97.3
TRY112	THR96	100.0
LEU122	MET106	100.0
SER128	ILE122	99.1
MET132	MET116	100.0
PHE134	LEU118	99.5
PHE136	PHE120	100.0
ILE157	ILE143	100.0
TYR159	TYR145	99.5
VAL183	ILE169	99.6^*a*^
ILE194	MET180	100.0^*a*^
VAL196	ILE182	100.0^*a*^
TYR205	TYR191	100.0^*a*^
HIS207	MET193	100.0^*a*^
PHE237	MET215	100.0^*a*^
LEU240	ILE218	100.0^*a*^
MET261	PHE239	99.5^*b*^

^*a*^ Indicates two public sequences with early truncation events, resulting in deletions at this residue.

^*b*^ Indicates three public sequences with early truncation events, resulting in deletions at this residue.

## DISCUSSION

This case highlights the potential severity of neonatal enterovirus and considerations in therapeutic decision-making. IVIG has been employed as a therapy for neonatal enterovirus, but the risks of volume overload appeared to outweigh the potential benefits for our patient (20). In addition, pocapavir is only available in an enteral formulation, which our patient could tolerate. We did not identify drug-related toxicity; notably, while his bilirubin rose following administration, previous studies have not identified elevated bilirubin, and we suspect that direct viral injury was the likely etiology (5, 6, 21).

Our decision to use pocapavir was based on the patient’s clinical severity. Following pocapavir, our patient improved with resolution of coagulopathy and liver dysfunction, but we observed little effect on CT values. We hypothesized that the lack of improvement could have been due to viral resistance or because pocapavir was initiated late in the course of illness (DOI 6), limiting its impact. Of note, while one prior report of enteroviral sepsis demonstrated a substantial increase in the CT (thus a decrease in viral load) within the first week of enterovirus illness (3), prolonged enteroviral persistence despite clinical improvement, as seen in our patient, has also been previously described (22). Thus, the observed persistence could represent the natural course of infection.

Susceptibility to pocapavir varies significantly between enterovirus serotypes and between isolates (15). Given the slow turnaround time of phenotypic resistance testing using viral culture, which limits its clinical utility, we explored whether resistance could be predicted via genomic sequencing. Our results show sequencing may have limited utility in predicting susceptibility. While we were able to identify the patient’s isolate as belonging to the B4 lineage, the mutations that contribute to pocapavir resistance in B4 strains are unknown, and *in silico* methods to predict resistance are limited. Phenotypic dose-response testing would therefore be required to determine isolate susceptibility but may be unfeasible to utilize, as this requires viral culture.

This case demonstrated a common challenge for clinicians in the treatment of neonatal enterovirus disease. While pocapavir appeared safe in early studies, this medication is not well studied in infants (6, 22). For our patient, the inability to use other potential therapies and his critical status suggested potential benefits outweighed possible risks. While viral sequencing may offer clues regarding response to pocapavir, limitations include turnaround time and a paucity of data to correlate genotype to phenotypic resistance.

## Data Availability

The pretreatment viral genomic sequence has been deposited in GenBank under accession number PV424085. The pre- and post-treatment Illumina sequencing reads have been deposited in the NCBI Sequence Read Archive (SRA) as SRR32923637, SRR32923636, and SRR32923635 under BioProject PRJNA1244348.
